# Cyclic Impact Compaction of an Ultra High Molecular Weight Polyethylene (UHMWPE) Powder and Properties of the Compacts

**DOI:** 10.3390/ma15196706

**Published:** 2022-09-27

**Authors:** Alexandr Shtertser, Boris Zlobin, Victor Kiselev, Sergei Shemelin, Arina Ukhina, Dina Dudina

**Affiliations:** 1 Lavrentyev Institute of Hydrodynamics, Siberian Branch of the Russian Academy of Sciences, Lavrentyev Ave. 15, 630090 Novosibirsk, Russia; 2 Design and Technology Branch of Lavrentyev Institute of Hydrodynamics, Siberian Branch of the Russian Academy of Sciences, Tereshkovoi Str. 29, 630090 Novosibirsk, Russia; 3 Institute of Solid State Chemistry and Mechanochemistry, Siberian Branch of the Russian Academy of Sciences, Kutateladze Str. 18, 630117 Novosibirsk, Russia

**Keywords:** cyclic impact compaction, explosive pretreatment, mechanical properties, nanoscale detonation carbon, ultrahigh molecular weight polyethylene

## Abstract

Experiments on Cyclic Impact Compaction (CIC) of UHMWPE powder GUR 4120 were carried out on a laboratory hydro-pneumatic impact device. This device provides impact energies of up to 1 kJ with a frequency of impacts of 9 s^−1^ and enables producing dense and robust compacts in the form of disks with a diameter of up to 60 mm and a height of up to 24 mm. The optimal parameters of the CIC were determined, which are the preheating temperature of the powder, the impact energy and the number of impacts. The strength, Brinell hardness and elongation of the resulting compacts with a diameter of 40 mm and a height of 15 mm were 37.5 MPa, 49.0 MPa and 470%, respectively. The possibility of activating UHMWPE powder by explosive loading was studied. It was found that the explosive pretreatment reduces the mechanical properties of the resultant compacts. The CIC method is suitable for the manufacture of UHMWPE-based composites with nano-additives, as evidenced by the successful production of compacts containing nanoscale detonation carbon as an additive. The results of the present study show that the CIC method is promising for the industrial production of small-sized UHMWPE parts.

## 1. Introduction

Ultrahigh Molecular Weight Polyethylene (UHMWPE) is a linear homopolymer. It has been widely used in industry and medicine since the 1960s [1,2]. UHMWPE is known for its high wear resistance, high toughness, high impact strength and low friction coefficient, as well as durability, biocompatibility and chemical inertness. These properties make UHMWPE the material of choice for a wide range of applications, such as chain guides, star wheels, truck beds, lead acid battery separators, as well as knee, shoulder and hip implants in modern medicine [2,3]. Owing to its high impact strength and toughness, UHMWPE is also used as armor protection [4,5]. 

UHMWPE is produced by polymerization of ethylene gas. Its large-scale industrial production in powder form became possible thanks to the process of metal-complex catalysis of olefin polymerization discovered in the 1950s by K. Ziegler and G. Natta [6,7]. However, at that time, the production of dense and durable bulk products from the powder by pressing, extrusion, injection molding, blow molding, screw extrusion or sintering, etc., was hampered by the high viscosity of the melt caused by its high molecular weight (more than 10^6^ g/mol) [8,9]. Therefore, the processing technologies of thermoplastics had to be significantly improved in order to adapt them to UHMWPE [8,9,10]. At present, the production technologies of bulk UHMWPE are still being developed [11,12,13,14,15,16].

Currently, the industrial production of bulk UHMWPE is associated with compression molding, ram extrusion, gel extrusion and spinning, etc. [1]. Usually, the as-polymerized UHMWPE powder is processed by ram extrusion or compression molding techniques at elevated temperatures (200–240 °C) and pressures (8–10 MPa). These processes are complex, expensive and time-consuming (may require several hours). In 2007, for the first time, a method of impact compaction of UHMWPE was proposed [17]. This method consists of applying a series of blows of an impactor to the powder enclosed in a metal mold (cyclic impact compaction). This technology enables producing small flat-shaped parts within several minutes. The mechanical characteristics of the bulk UHMWPE obtained by this method are comparable to those of the commercial UHMWPE products.

A further development of the cyclic impact compaction (CIC) method was presented in [18,19,20]. In 2004, the possibility of shock-wave compaction (a compaction method based on the use of high explosives) of UHMWPE powders was reported [21]. Compacts obtained by shock-wave compaction required further processing by sintering to achieve the desired mechanical properties. It should be noted that coatings formed from UHMWPE are also of interest [22,23].

The properties of UHMWPE can be modified by introducing additives in the polymer matrix. Additives in the form of carbon or glass fibers [24,25,26], nanoscale carbon [27,28], and different other substances [29,30,31,32] have been used for this purpose. 

In this paper, the possibilities of the CIC method to produce compacts from UHMWPE and mixtures of UHMWPE and nanoscale detonation carbon (NDC) are presented. The optimal conditions for the manufacture of the compacts are reported.

## 2. Materials and Methods

The UHMWPE powder from which the samples were made by the CIC method was GUR 4120 (Ticona GmbH, Frankfurt, Germany) with a particle size of 120–140 μm and molar mass of 5·10^6^ g/mol. The CIC method was described in our previous works [18,19,20], where the results of producing compacts from pure UHMWPE GUR 4150, as well as UHMWPE-based composites with micro- and nano-additives and with reinforcing metal inserts are presented. The additives used in [18] were TiO_2_ (brand R-FC5), SiO_2_ (Rosil-175 silica filter) and technical SiC powders with an average particle size of 180 nm, 17 nm and 10 μm, respectively. In this work, we used as an additive nanoscale detonation carbon (NDC) whose production method and properties are described in [33,34,35]. NDC is the product of the decomposition of acetylene as a result of the detonation of the acetylene–oxygen mixture C_2_H_2_ + *k*O_2_ with a low oxygen content (*k* < 1). It has low bulk density varying from 0.02 to 0.05 g/cm^3^, depending on *k*, and contains particles both round, tens of nm in size and graphene-like, 100–200 nm in size.

**Figure 1:**
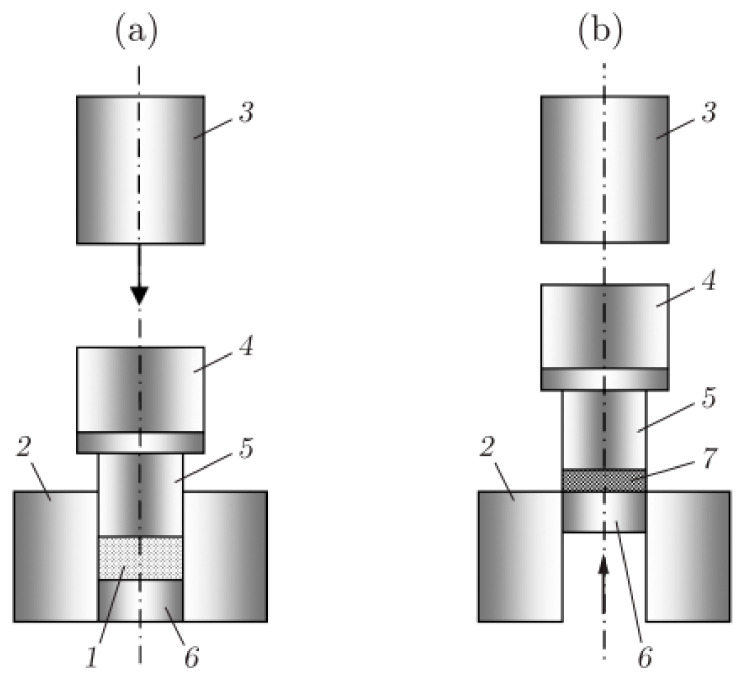
The principle of the CIC method: (**a**) position of the apparatus during compaction; (**b**) position of the apparatus after the end of compaction; (1) pressed powder; (2) die; (3) impactor; (4) spacer; (5) mushroom-shaped piston; (6) ejector; and (7) prepared compact. Reprinted with the permission of the Journal of Applied Mechanics and Technical Physics from [18]. Copyright 2017.

**Figure 2:**
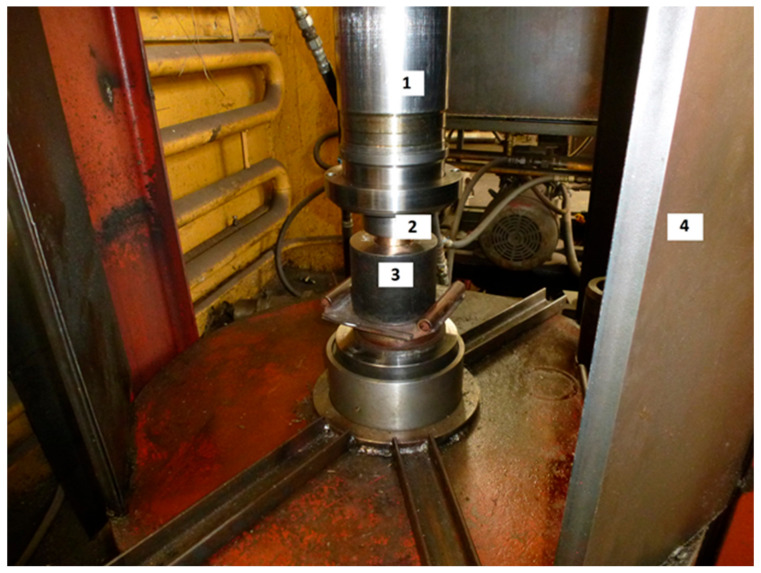
Hydro-pneumatic impact device for compaction of UHMWPE powder: 1, impactor; 2, piston; 3, die; 4, device housing.

Figure 1 illustrates the principle of the CIC method. It is based on the use of a hydro-pneumatic impact device designed at the Design and Technology Branch of Lavrentyev Institute of Hydrodynamics (DTB of LIH) for briquetting powder materials and which is shown in Figure 2. 

The facility enables striking the processed material with a frequency of 9 s^−1^ and an impact energy of up to 1 kJ. Additionally, it is possible to create a static force of 4 tons for pre-pressing the processed powder. According to [17,18], the UHMWPE consolidation process during shock treatment consists of two stages. At the first stage, after a certain number of impacts, the material is compacted to almost zero porosity; at the second stage, melting occurs at the boundaries of the particles, and their welding as a result of recrystallization during cooling. This consolidation process is similar to the processes proceeding during shock wave loading of metal and ceramic powders described in detail in [36].

The pressing procedure consisted of the following steps:

(1) Holding the powder under static pressure of the impact press (5–10 s) after filling into the mold;
(2) Heating the mold with the powder to a predetermined temperature;
(3) Carrying out CIC;
(4) Cooling the sample in the mold under the static pressure.

The main parameters of the CIC process are the preheating temperature of the mold with UHMWPE powder, the impact energy and the number of blows applied. In order to determine the optimal compaction mode during the studies, the preheating temperature was varied from 80 to 140 °C, the impact energy from 196 to 983 J and the number of impacts from 2000 to 5000 (CIC processing time 4–10 min). The resulting samples were disks with a diameter of 40 and 60 mm, and the thickness varied from 11 to 30 mm. The impact energy (kinetic energy of impactor *3* in Figure 1) depends on the air pressure in the pneumatic system of the impact device, so that the pressure of 0.3, 0.5, 0.7, 1.0 and 1.5 MPa corresponded to the impactor kinetic energies of 196, 330, 460, 655 and 983 J.

The produced samples were studied by optical microscopy using an OLYMPUS GX-51 microscope (Tokyo, Japan), measurements of hardness using a DuraScan-50 device (EMCO-TEST, Kuchl, Austria) and 2137 TU hardness tester, and measurements of tensile strength and elongation using a ZDM-5 testing machine. The X-ray diffraction (XRD) patterns of the initial powders and compacts were recorded by means of a D8 ADVANCE powder diffractometer (Bruker AXS, Karlsruhe, Germany) with Cu Kα radiation. 

**Figure 3:**
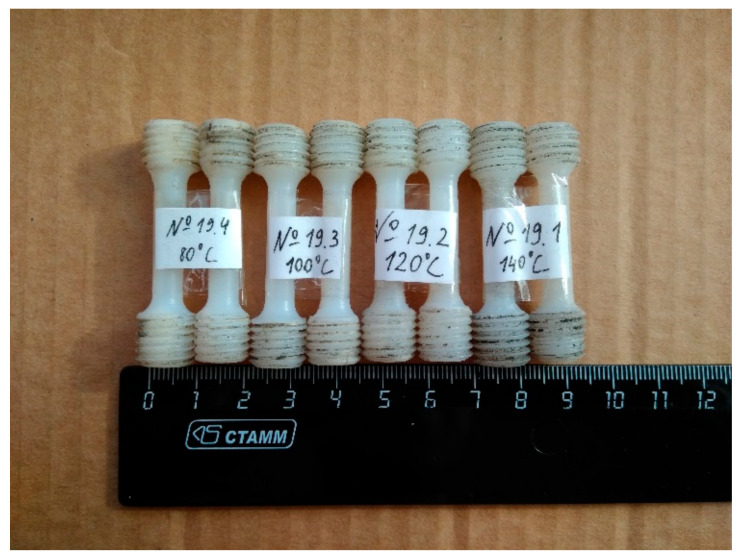
Tensile test samples made from compacts produced by CIC at preheating temperatures of 80, 100, 120 and 140 °C.

Figure 3 shows tensile test samples cut from compacts with a diameter of 60 mm made at different heating temperatures of powder before CIC. Samples were cut along the diameter of the compacts.

**Figure 4:**
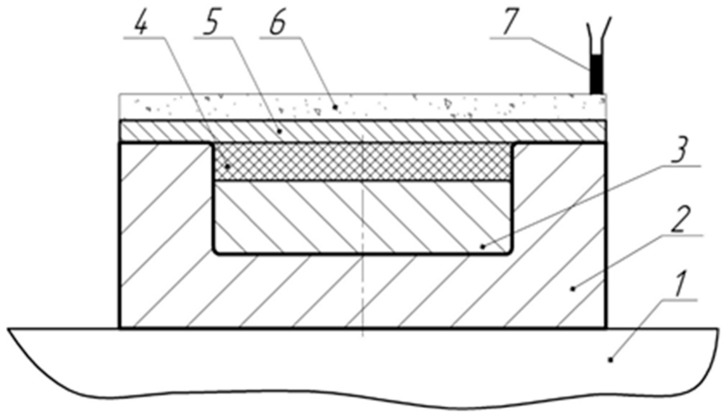
Powder activation scheme by explosive loading: 1, explosion chamber desktop; 2, mold; 3, insert; 4, UHMWPE powder; 5, steel plate (7 mm thick); 6, high explosive; 7, high-voltage detonator.

**Figure 5:**
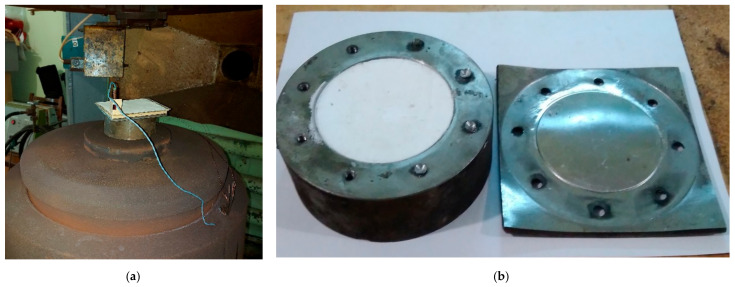
Powder activation by explosive loading: (**a**) experimental assembly mounted on the table of an explosion chamber with HE charge on top; (**b**) mold with treated powder after removing the steel plate.

Some researchers point out that the preliminary explosive loading of UHMWPE powder before sintering improves the properties of the resulting bulk material [21]. We also decided to check whether it is possible to activate this polymer with a shock wave excited by the detonation of high explosive (HE) before making a compact by the CIC method. To do this, the powder was placed into the steel mold and covered with a steel plate, which was bolted to the mold. The mold had an outer diameter of 130 mm and a height of 50 mm. The cavity for placing the powder in the mold had a diameter of 80 mm and a depth of 12 mm; the steel plate had dimensions of 130 × 130 × 7 mm^3^. HE was placed on top of the plate, and an explosion was initiated, as shown in Figure 4 and Figure 5. 

Blasting operations were carried out in the KV-2 explosion chamber, designed and manufactured in DTB of LIH. This equipment is designed for a working charge of 2 kg of HE in TNT equivalent.

The HE in the experiments were amatol and mixtures of RDX with baking soda (NaHCO_3_). The dimensions of the HE flat charge were 130 × 130 × 6 mm^3^ for amatol and 130 × 130 × 5 mm^3^ for mixtures of RDX with soda. After the explosion, the bolts were unscrewed, the plate was removed, and the shock wave-treated powder was extracted from the mold. Thereafter, compacts with a diameter of 40 mm were made from the processed powder by CIC in another mold.

**Figure 6:**
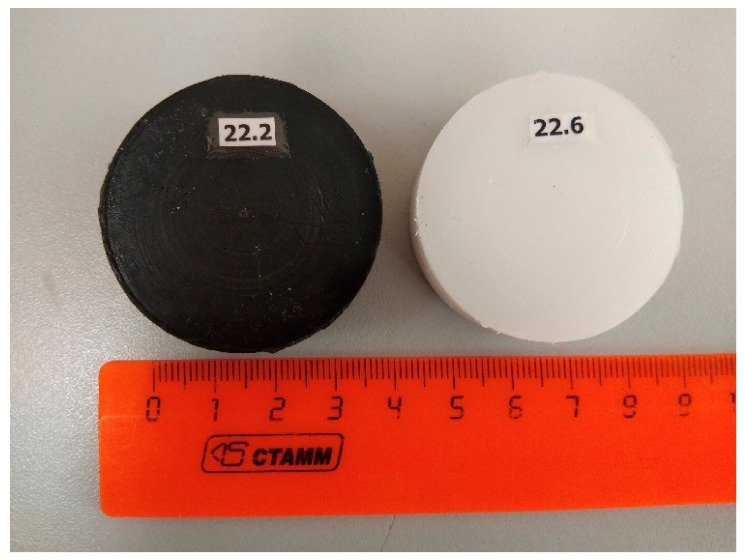
Compacts with a diameter of 40 mm and a height of 15 mm made of pure UHMWPE (**right**) and UHMWPE + 0.5 wt.% NDC (**left**).

Compacts with the addition of NDC were made as follows. First, there was a procedure for preparing a mixture of UHMWPE powder with NDC. It includes placing the UHMWPE powder in a glass cup, adding ethyl alcohol (5–10 mm above the powder level), adding the required amount of NDC and thoroughly mixing the formed mass to a homogeneous state. Next, the cup was placed in a drying oven and kept at a temperature of 80 °C until the alcohol completely evaporated. A cup with a capacity of 150–250 mL usually requires about two hours of exposure in the oven. After this procedure, sieving or grinding of the resulting powder mixture is not required. The prepared mixture was compacted by the CIC technique. The resulting compacts are black in color; Figure 6 shows compacts made under the same conditions from a pure polymer and from a polymer with an additive of 0.5% NDC. 

## 3. Results and Discussion

First, we will make a remark about the accuracy of the data below on the strength characteristics of the compacts made of UHMWPE. The data presented are the average values obtained by measurements of several samples. The spread (standard deviation) of the values of strength, elongation and hardness on different samples made under the same conditions did not exceed 10–12%. The density of the compacts was determined with an accuracy of ±0.01 g/cm^3^.

**Figure 7:**
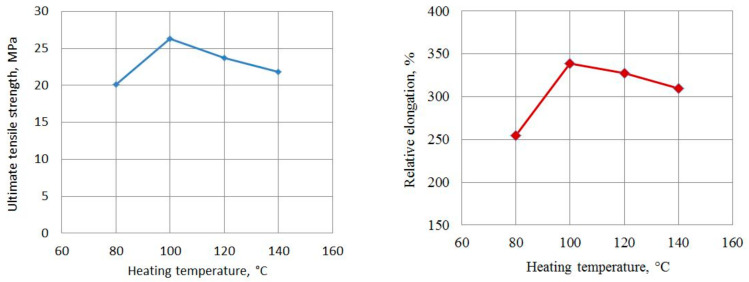
Dependence of the strength (**left**) and the relative elongation (**right**) of the UHMWPE compact on the preheating temperature of the powder.

Figure 7 shows the dependences of the tensile strength and elongation of compacts on the preheating temperature of the powder. The duration of compaction at all temperatures was 200 s, and the impact energy was 983 J. Both dependences have a maximum at *T* = 100 °C, and this temperature is most likely optimal for CIC. At higher temperatures, there is a decrease in the strength of the material, which may be associated with an increase in the proportion of the amorphous phase in the volume of the resulting material when it is overheated. It is known that to obtain a durable material in the technology of static pressing, the heating temperature of the UHMWPE powder usually is 200–240 °C, while in the CIC process, as we see, the optimal temperature is much lower. In our opinion, this is due to the fact that when compression waves pass through the processed powder, the temperature at the contacts of the particles due to friction increases more than in their volume. The issues related to inhomogeneous heating of powder particles under dynamic loading are well described in [36]. If the powder is preheated to a sufficiently high temperature, then during the passage of compression waves, the particles at the boundaries melt. With further cooling, recrystallization occurs, and a strong bond is formed between them. 

If the preheating temperature is low, then the heating of the polymer during a series of impacts does not lead to its complete melting over the entire contact surface of the particles, so the strength of the compact decreases. Thus, the diagrams in Figure 7 show that heating up to 100–110 °C is optimal in the manufacture of compacts from UHMWPE. In the range from 100 to 110 °C, the strength drop is not more than 5%.

Another important parameter from the point of view of industrial application of the CIC method is the impact energy, by which we mean the kinetic energy of impactor *3* (see Figure 1). At first glance, it seems that the greater the impact energy, the stronger the compact should be. However, with an increase in energy, the elements of the impact device experience higher and higher loads, which leads to an increase in breakdowns of this device. If breakdowns become too frequent, the industrial application of the CIC method may become inappropriate. In addition, an excessively high impact energy can lead to the appearance of defects inside the particles and to the destruction of already formed bonds between the particles. We most likely observe such a situation when loading the UHMWPE powder with shock waves excited by an explosion, as will be discussed below. Table 1 shows data on the tensile strength of compacts obtained with different impact energies. The steel impactor in the impact device has a diameter of 80 mm, a length of 840 mm and a mass of 33 kg. In the last column of the table, there is no data on the strength of samples 19.5 and 19.6 because the strength of the compact was so low that the samples broke during their manufacture.

It follows from Table 1 that as the impact energy increases, the strength of the compact also increases. However, as experiments have shown, at an energy of 983 J, the load on the elements of the impact device becomes too high; some parts often fail and require replacement. In our opinion, from the point of view of the operability of the impact device, the impact energy of 900 J, at which the experiments described in [18] were carried out, can be recommended as the maximum for compacting the UHMWPE powder. Since, according to Table 1, the strength of the compact still remains quite high at an impact energy of 655 J, this energy can be recommended as the minimum for compacting this powder by the CIC technique.

Another important technological parameter is the number of strokes made during the manufacture of the compact. Table 2 provides information on compacting UHMWPE powder with a different number of impacts. The preheating temperature of the powder was 100 °C; the impact energy was 655 J. As can be seen from this table, an increase in the number of impacts from 2000 to 5000, i.e., by 250%, leads to an increase in the strength of the compact by only 13.5%. However, at the same time, the duration of the manufacturing cycle for one compact increases significantly, i.e., productivity decreases.

It can be assumed that an increase in the plasticity of the compact with an increase in the total energy of impacts is associated with a decrease in the content of defects in the form of micro- and nanopores in its volume. An increase in the number of impacts leads to a more complete displacement of air and the closure of small pores due to the localization of deformation at the contacts of particles. However, this assumption needs to be verified by a thorough study of the microstructure of compacts.

Thus, summing up the above data, the following technological parameters can be recommended for the production of bulk products from UHMWPE powder by the CIC method with the frequency of strokes 9 s^−1^: the energy of one stroke from 655 to 900 J, the preheating temperature of 100–120 °C and the total number of strokes from 2000 to 3000. Based on the data provided and on the results described in [18,19,20], it can be stated that the described CIC technology is suitable for the manufacture of UHMWPE parts with a diameter of up to 60 mm, a height of up to 24 mm and a weight of up to 65 g. Depending on the diameter of the compact, the recommended parameters of the CIC technique provide the tensile strength of compacts from 20 to 45 MPa and elongation from 185 to 470%. These characteristics, especially for samples with a diameter of 40 mm, are very close to the properties of UHMWPE compacts produced by the CIC method in the work [17] and by industrial methods of compression molding, ram extrusion and gel extrusion and spinning described in [1]. According to [1], samples obtained by industrial methods have a tensile strength of 41–44 MPa and a relative elongation of 350–450%. 

Table 3 presents the results of experiments on explosive loading of UHMWPE powder with its subsequent compaction by the CIC method. The characteristics of the used HE charges are also indicated there; amatol and mixtures of RDX with baking soda (NaHCO_3_) were employed as explosives. After explosive loading, the powder particles in some places stick together into small conglomerates, which easily fall apart. For comparison, the last row of Table 3 shows the properties of compacts made from non-activated powder. Apparently, the activation of the powder by explosion leads to worsening of the properties of the resulting compact. 

**Figure 8:**
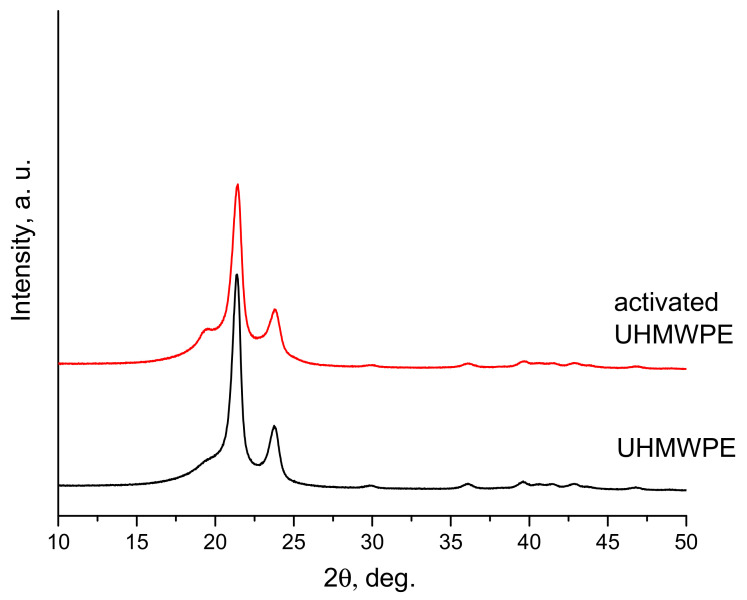
XRD patterns of the activated (top) and non-activated (bottom) UHMWPE powders.

In our opinion, the reason is that explosive loading leads to an increase in the amorphous component in the treated powder. As indicated in [17], the density of the amorphous phase in the polymer is *ρ_a_* = 0.855 g/cm^3^, and of the crystalline one is *ρ_c_* = 0.999 g/cm^3^. Therefore, the greater the content of the crystalline phase in the resulting material, the greater its density. As shown in [18], the volume fraction of the crystalline phase in the UHMWPE compact is determined by the expression *ν_c_* = (*ρ* − *ρ_a_*)/(*ρ_c_* − *ρ_a_*9), where *ρ* is the measured density of the compact. From Table 3, we see that the density of compacts made from activated powder is 0.92 g/cm^3^, while from non-activated powder, it is 0.93 g/cm^3^. Accordingly, the volume content of the crystalline phase is 45 and 52%, respectively. The fact that the shock wave caused by the explosion leads to an increase in the content of the amorphous phase is also confirmed by the XRD diagram shown in Figure 8. The two sharp peaks in the diagram correspond to the orthorhombic crystalline phase, and the step-shaped ledge to the left of the main peak is more associated with the contribution of the amorphous phase. Figure 8 shows that this ledge is more clearly expressed in the activated powder.

**Figure 9:**
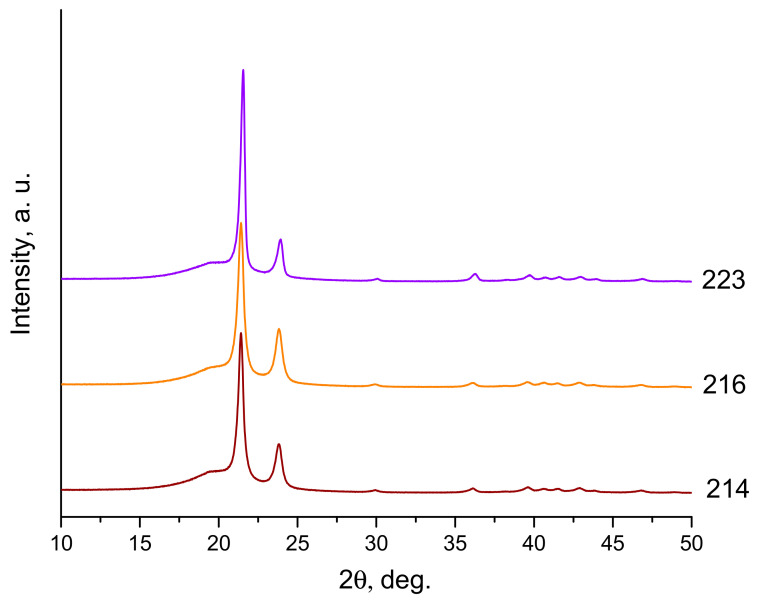
XRD patterns of compacts made by the CIC method from activated (214) and non-activated (216) powders and from a mixture of UHMWPE + 0.3 wt.% NDC (223).

Figure 9 shows XRD diagrams of compacts made from explosion-activated (curve 214) and non-activated (curve 216) powders, as well as a compact made from UHMWPE with an additive of 0.3 wt.% NDC (curve 223). As can be seen, the reflexes of the crystalline phases become narrower after CIC, and the content of the amorphous phase decreases.

Based on the above data, it can be stated that intensive shock-wave loading of the UHMWPE powder leads to its amorphization, while cyclic impact compaction, on the other hand, leads to an increase in the crystalline phase.

**Figure 10:**
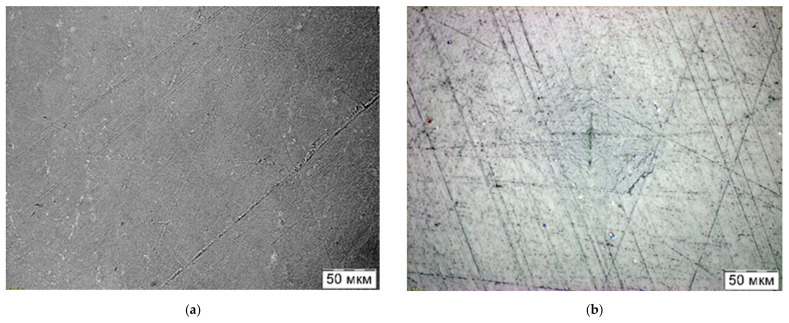
Microstructure of compacts made from the UHMWPE + 0.5 wt.% NDC mixture (**a**) and from pure UHMWPE (**b**).

The first trial experiments carried out in this work on the manufacture of a UHMWPE + NDC composite material (see Figure 6) showed that the CIC technology has the ability to produce composites based on UHMWPE relatively simply with the addition of nanoscale carbon. The method of mixing UHMWPE and NDC powders described in Section 2 enables having a homogeneous initial mixture and, accordingly, a homogeneous structure in the resulting compact. It follows from Figure 9 (curve 223) that the addition of nanoscale carbon does not lead to any significant changes in the polymer structure. The microhardness HV_0.01_ of the compact with the addition of NDC was 34.3 MPa, which is somewhat less than the hardness of the compact made of pure UHMWPE (38.3 MPa). Presumably, the addition of NDC does not worsen the strength characteristics of the polymer compact, although additional tests are required to be carried out, which are currently being planned. Figure 10 shows the microstructure of UHMWPE compacts with and without the addition of NDC. The structure of the compacts is nonporous, which proves that the CIC method provides complete removal of air from the pores at the initial stage of pressing and further consolidation of polymer particles.

Since pure UHMWPE is a dielectric with a volume resistivity of more than 10^12^ Ω·m [1], NDC additives can be useful when it is necessary to give electrical conductivity to a material. Measurements of the electrical resistance of compacts show that the addition of NDC to the polymer in an amount of 0.5 wt.% reduces the resistivity to 2.5·10^4^ Ω·m.

In our opinion, the technology considered in this article has limitations on the size of manufactured parts. For the manufacture of compacts weighing up to 65 g, a laboratory impact device with a plan size of 1.37 × 1.37 m^2^ and a height of 2.41 m was used. Obviously, an increase in the size and weight of manufactured parts will require a proportional increase in the size of the equipment. At the same time, the frequency of impacts may decrease, and accordingly, the time to manufacture the part will increase. The described experiments show that the CIC method is promising for the industrial production of small-sized UHMWPE parts. We believe that this technology can be effective in the industrial production of small parts, such as sliding bearings, parts of devices operating at low temperatures and, possibly, medical implants. Regarding implants, our preliminary experiments have shown that in the process of CIC, metal elements can be strongly in-pressed into a UHMWPE compact [19]. Herewith, the sealing strength is so high that when a steel rod with a diameter of 5 mm is pulled out of the compact, this rod breaks but cannot be removed from the compact. It is possible that a composite based on UHMWPE with the addition of NDC can be used in medical applications, since the latter has recently shown its biosafety in human cell culture [37]. 

## 4. Conclusions

In the conducted research, the optimal modes of producing compacts from UHMWPE powder by the method of Cyclic Impact Compaction (CIC) were determined. On a laboratory hydro-pneumatic device with an impact frequency of 9 s^−1^, it is possible to produce compacts with a diameter of up to 60 mm, a height of up to 24 mm and a weight of up to 65 g. The optimal preheating temperature of the powder before CIC is 100–110 °C. The number of impacts in the range of 2000–3000 with an impact energy in the range of 655–900 J provides sufficiently high strength properties in the resulting compacts. The upper limit of the impact energy is caused by the protection of the equipment used from frequent breakdowns under the effect of shock loads and possibly can be increased by further improving the design of the hydro-pneumatic impact device. In contrast to the works in which the possibility of UHMWPE activation by explosion is noted, this work shows that pretreatment of the UHMWPE powder by explosive loading leads to a decrease in the strength properties of the resulting compacts. This decrease is most likely due to an increase in the proportion of the amorphous phase in the polymer after explosion treatment. It is shown that the CIC method enables producing a composite based on UHMWPE in a relatively simple way with the addition of Nanoscale Detonation Carbon (NDC) produced by detonation decomposition of acetylene. In our opinion, further research should focus on obtaining composites based on UHMWPE with NDC as an additive, since this material is new and insufficiently studied as a component of composites. 

## Figures and Tables

**Table 1:** Parameters of compacts made at different kinetic energy of the impactor.

Sample Series Number	Air Pressure in a Pneumatic System, MPa	Impactor Kinetic Energy, J	Compact Parameters			CIC Processing Duration, s	Tensile Strength, MPa
			Height mm	Mass, g	Density, g/cm^3^		
19.5	0.3	196	15.8	41.9	0.92	333	-
19.6	0.5	330	15.4	41.7	0.94	286	-
19.7	0.7	460	15.2	41.7	0.95	250	13.0
19.8	1.0	655	15.0	41.1	0.95	222	20.0
19.9	1.5	983	15.2	40.8	0.96	200	26.8

Notes: the diameter of the compacts varied in the range of 59.7–60.7 mm; the blows followed with a frequency of 9 s^−1^.

**Table 2:** Compacting UHMWPE powder with a different number of impacts.

Sample Series Number	CIC Processing Duration, s	Number of Impacts	Total Impact Energy, MJ	Tensile Strength, MPa	Relative Elongation, %
19.8	222	2000	1.31	20.0	185
19.10	336	3024	1.98	20.9	255
19.11	556	5000	3.28	22.7	295

**Table 3:** Parameters of UHMWPE powder activation by explosion and properties of compacts made from activated powder by CIC: *h*_e_, the thickness of the HE charge; *D*, the detonation velocity of HE; *P*, pressure behind the detonation front; *ρ*, compact density; *σ*, compact tensile strength; *δ*, elongation; HB, compact Brinell hardness.

Sample Series Number	HE	*h*_e_, mm	*D*, km/s	*P*, GPa	*ρ*, g/cm^3^	*σ*, MPa	*δ*, %	HB, MPa
21.1	Amatol	6	2.36	1.43	0.92	25.8	390	56.8
21.2	RDX + soda, 1:1	5	1.95	1.51	0.92	28.1	430	50.2
21.3	RDX + soda, 2:1	5	3.16	3.45	0.92	27.4	430	46.7
21.4	RDX + soda, 3:1	5	3.9	4.87	0.92	26.9	430	49.0
21.6	-	-	-	-	0.93	37.5	470	49.0

Notes: the diameter of the compacts varied from 40.0 to 41.0 mm, height from 15.1 to 16.3 mm; samples 21.6 were made from non-activated powder; the mass of the HE charge from amatol was 90 g, from mixtures of RDX with soda 85 g.

## Data Availability

Not applicable.
